# Understanding Users in the ‘Field’ of Medications

**DOI:** 10.3390/pharmacy4020019

**Published:** 2016-05-06

**Authors:** Peri J. Ballantyne

**Affiliations:** Department of Sociology, Trent University, 1600 West Bank Drive, Peterborough, ON K9J 0G2, Canada; periballantyne@trentu.ca; Tel.: +1-705-748-1011 (ext. 7813); Fax: +1-705-748-1213

**Keywords:** social pharmacy, medicine use, medicine user, field of medications

## Abstract

The numbers of medicinal drugs available for human consumption have increased rapidly in the past several decades, and physician prescribing practices reflect the growing reliance on medicines in health care. However, the nature of medicines-as-technology makes problematic taken-for-granted relationships among actors involved in the delivery, or who are the recipients of medicines-reliant health care. In this article, I situate the medicine user in the ‘field’ of medications—where interests, actions and outcomes are continually negotiated among and between the various players—physicians, pharmacists, government regulatory bodies, the pharmaceutical industry and users of medicines. The objective of the paper is to illuminate the complex context in which the medicine-user—the target of the pharmacy profession’s service to the public—accesses and uses medicines.

## 1. Introduction

In the past several decades, medicines have come to occupy an increasingly important place in health care practice. To get a sense of the scope of this phenomenon, consider that, in Canada in 2000, there were an estimated 22,000 (excluding biologic drug products, controlled substances and complementary and alternative health products ) medicines available for human consumption—about 5200 of which were prescription medicines [1] (p. xxxii). At that time, it was noted that 300 million prescriptions were being filled in Canada each year—an average of ten per person [1] (p. 191). Globally [2], and in developed nations such as Canada [3], the UK [4], and the US [5], trends of increasing reliance on pharmaceuticals in health care, reflected in pharmaceutical sales, pharmaceutical expenditures, and in the numbers of prescriptions dispensed, are evident.

I use the concept of ‘medicines proliferation’ to refer to this rapid rise in the production, marketing and application or use of medicinal drugs for the production of health and the prevention and treatment of illness. Medicines proliferation has emerged in a neo-liberal political environment in which health and health care is individualized and de-politicized [6,7]; and where the pursuit of health is driven by health consumers who make both strong demands of conventional medical practice and challenge its limitations [8]. Representing an internalized sense of responsibility for one’s health, or *healthism* [9,10], in a neoliberal context, citizens engage in a kind of ‘government of the self’ [11], involving the surveillance of themselves, and the consumption of medical services and products in a quest to optimize their productivity and functionality.

The application of Foucault’s idea of self-governance is particularly suitable for examining users’ negotiations of medicines [12]. This is because medicines proliferation has emerged in an environment where, increasingly, medicines are provided in ambulatory and community settings. This has the consequence of restricting the capacity of health professionals such as physicians and pharmacists to monitor medication use and effects; and of locating the control of pharmaceuticals in the hands and homes of users [13,14]. Thus, while physicians (and pharmacists) control users’ *access* to prescription medicines, they do not control their uses once a prescription is issued (with some exceptions [15]. Moreover, the vast majority of medicinal products, including natural health products sold in many pharmacies, do not require authorization from a prescriber, and are available to anyone with the resources to purchase them. Thus, the social context of medicine-use is broad and complex, straddling sites of organized health care as well as the everyday settings of users’ homes and communities [14]. To consider how this diverse and dispersed context influences medicine use, Bourdieu’s concept of the ‘social field’ proves useful.

## 2. The Field of Medications and Its Agents

Bourdieu’s ‘field theory’ is a theory of social class and of the structured relations among actors in a class system. Bourdieu conceptualized the field as a social space in which actors and agents interact and compete for social position and authority based on their shared interest in the objects in the field, and their access to different forms of capital—or resources [16]. Bourdieu conceived of several forms of capital: economic—referring to monetary income and other financial resources; cultural capital including resources in the form of education and knowledge; social capital refers to the actual and potential resources available through membership in social networks and organizations. Symbolic capital refers to the capacity to define and legitimize cultural, social and moral values [16] (p. 862).

**Figure 1:**
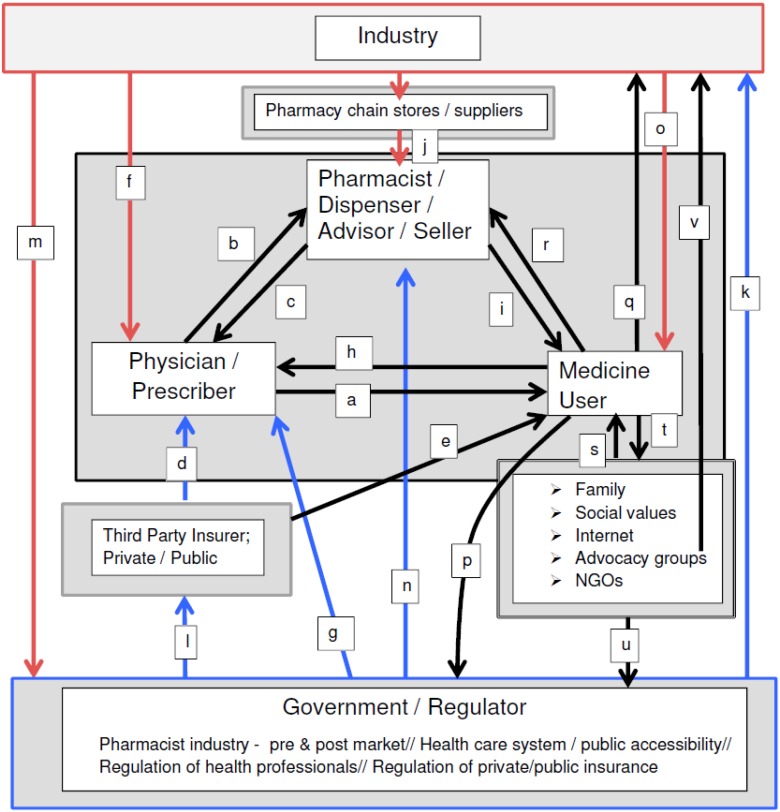
Relationships among Agents in the Field of Medications.

While Bourdieu’s theory has been applied previously in research analyzing older people in the ‘field of medications’ [17], in this article, I consider the collective set of actors and activitiesrelated to the development, exchange and uses of medications in this field. The major players (actors or agents) sharing an interest in the field of medications—and wielding different forms of capital—include physicians, pharmacists, governments, the pharmaceutical industry—and users of medicines. Physicians are the gatekeepers to prescription medicines, while pharmacists hold knowledge capital and certain legal controls over pharmaceuticals (*i.e.*, point of sale). Governments that create and regulate health and pharmaceutical policy and pharmaceutical manufacturing, and the manufacturers producing medicinal products are among other agents who wield influence in this field. Users—arguably the most important agent in the field—are influenced by the interests and actions of other players; and they also exert influence in the field. In the remainder of this article, I discuss these key agents/actors—and their interests, actions and impacts that—under continual negotiation with other players—influence the complex and evolving social context in which individuals receive and use medicines. The relationships between and among specific agents in the field of medications are outlined in Figure 1; alphabetic notation indicates specific relationships, as discussed herein.

### 2.1. Physicians

Representing the profession of medicine, physicians hold social, cultural, economic and symbolic capital. Medicine’s legitimacy over defining and treating health and illness has traditionally been based on knowledge claims about progressive forms of health care and the value of emerging technologies such as new medical drugs [4,18]. Physicians’ capital is based on legislation, education, and high public trust and recognition. Physicians are the gatekeepers to formal conventional health care, and in Western countries, to prescription medicines; and they influence the public’s choices and uses of non-prescription medicines.

The profession’s explicit interest is in achieving optimal outcomes pertaining to patient care (see Figure 1a) [19]. To achieve these outcomes, physicians interact with patients and allied health professionals, including pharmacists (see Figure 1b)—the latter group providing an important check and balance role *vis-à-vis* a physician’s recommended treatment plan (see Figure 1c). An optimal scenario in the field of medication is one where the physician sees the patient in timely manner, makes a correct assessment and diagnosis, and recommends the optimal medication, which results in a positive health outcome for the patient. However, physicians’ interactions with other actors in the field impact their prescribing practices, and thus, their relationships with patients or prospective patients, and with patient outcomes.

Physicians’ treatment choices are typically restricted to what has been negotiated by public or private insurance programmes for medical care and for medicines—the third party payers in the field of medications (see Figure 1d). Third party payers are motivated to remain solvent and be accountable to the tax-payer (in publicly insured health care) and to produce profits (in the private insurance industry). Third party payers effectively use various ‘demand control strategies’ to discipline users’ access to medicines [20] (Figure 1e). A consequence is that physicians’ treatment recommendations may be restricted and their desired or optimal choices for a patient may be unavailable [19].

In a context of governance favouring its interests, the pharmaceutical industry has been highly successful at leveraging the medical profession’s symbolic and social capital in both ‘upstream’ and ‘downstream’ processes of pharmaceutical development and distribution [21]. For example, manufacturers seek medical leaders’ involvement in the development and testing of new medicines [22], and in the funding and publication of clinical trial results [23]. They influence physicians’ prescribing through academic detailing efforts made by pharmaceutical representatives [24,25,26], and undertake other creative endeavors [27] so as to influence physician prescribing (Figure 1f).

Regulatory bodies’ safety and efficacy standards for the release a new drug to the market also impact physician prescribing by determining the quality of medicines available [28,29]. Un-discovered or un-reported risks [30,31,32], as well the availability of medicines with known risk profiles [33] may result in poor medication-use outcomes for some patients, despite a physician’s best intent. And, while drug approval is regulated, physician prescribing of a regulated drug is not: liberal scope of practice legislation enables widespread ‘off-label’ prescribing practices that impact patient outcomes in positive as well as negative ways [34,35] (Figure 1g).

Finally, physicians are influenced by the public and by patients or potential patients in several ways. As active, reflective consumers and ‘expert patients’, members of the public evaluate and have the potential to accept, challenge or resist the profession’s claims about the value of medicines [2,21]. People’s beliefs about medicines, their direct or vicarious experiences of them, their attention to or knowledge of documented side effects and risks associated with specific medicines—all influence their interests in and alignment with medical opinion or an individual physician’s medication recommendations [13,36]. In addition, physicians have been shown to respond to patients’ medication expectations or demands for a prescription, even if these contradict the physician’s intended actions [37] (Figure 1h).

Hence, while seemingly autonomous actors, physicians are influenced by various other agents, whose interests in the field of medicines may align or conflict with the explicit intention a physician has for a patient.

### 2.2. Pharmacists

Pharmacists occupy a role supportive of physician-prescribers, and are vested with the responsibly of circumventing medication errors and drug-related problems, and counseling and educating patients around the appropriate use of their prescribed medicines. In this way, pharmacists reinforce the symbolic, social and economic capital of the physician. However, pharmacists also independently wield these forms of capital. Pharmacists have power over dispensing and distributing pharmaceuticals (selling and profiting from the sales), and they have educational capital.

Pharmacists are required by law to be present at the site of prescription dispensing and sales and they also mediate (much of) the public’s access to over-the-counter or non-prescription medicines. Being widely accessible and visible in communities, pharmacists are sought out by (and advertise themselves to) the public as the legitimate advisors for the self-management of prescription medicines, and self-care using over-the-counter medicines [38]. Pharmacists have actively sought out an expanded role in public health [39,40,41], claiming education/knowledge capital and accessibility to the public. The profession espouses the practice philosophy of ‘pharmaceutical care’ [42]—or in the UK, ‘medicines management’ [43]—that aligns with medicine’s ‘patient-focused’ care philosophy [44] characterized by professed fidelity to patient- over commercial- or other-interests (Figure 1i).

A primary barrier to broad public endorsement for an expanded role for pharmacists in public health is “the contradiction of attempting to graft a public health mindset onto a commercial environment” [43] (p. 167). The corporatization of community pharmacy—involving the community pharmacist’s displacement from traditional, independent pharmacy shops to large, independent chain-store pharmacies where they are subjected to de-skilling and the routinization of activities in place of enacting autonomous professional services to the public [45,46]—starkly illuminates this key dilemma (Figure 1j). The public’s perspectives on the pharmacy profession’s expanded role in public health will be revealed in its actions in seeking primary health care in the community pharmacy setting [47]. In the community—where most are employed—pharmacists seeking recognition as public health professionals may be subjected to more intense public scrutiny and skepticism than physicians, given that their remuneration is primarily tied to selling medicinal products rather than cognitive health care services [43].

### 2.3. Government Regulation in the Field of Medications:

Governments are also positioned with bifurcated interests in medicines-related health care. On the one hand, from the 1920s to the mid-1970s, all Western industrialized countries introduced government regulation of drug safety and efficacy, and “for the first time, only government agencies had the legal authority to determine whether a new drug was safe and effective enough to be permitted on the market” [28] (p. 872) (Figure 1k). In addition, over the same time—in contexts such as in public health care systems—governments have established processes for reviewing clinical and economic data necessary for evidence-based drug coverage policy [48] and have implemented other strategies to regulate or negotiate the costs of medicines (*i.e.*, Canada’s Patented Medicines Prices Review Board [49], the UK Pharmaceutical Price Regulation Scheme [50], and the US Medicare Prescription Drug Price Negotiation Act of 2007 [51] (Figure 1l). In this way, governments hold a form of symbolic capital that highlights the responsibility to promote and protect the public’s interests in having safe, effective and affordable medicines on the market.

On the other hand, governments have an interest in the pharmaceutical economy and its promotion—and have capitulated in favour of the industry’s interests in the drug regulatory process, specifically regarding expedited pre-market evaluation of new medicines [28,29], and in Europe, in the post-license marketing context, enhanced freedoms to provide product information and education to the public [52,53]. Critics have suggested that these changes have emerged, in part, because over the past couple of decades, public funding of government regulatory bodies has been supplanted by funding via drug company fees. For example, in Canada, by 1998, cost recovery from manufacturers was reported to cover 75% of the required budget; effectively creating a regulatory ‘user pay’ system that has been oriented away from serving the public as client to serving the industry as client [29]. Similar changes have occurred in the US and in European Union countries [29]. In the UK, this has been characterized as corporate bias, where:

“the pharmaceutical industry was and is permitted to have strategic access to, and involvement with, government regulatory policy over and above any other interest group; and more often than other factors, the industry was, and is, decisive in determining regulatory policy outcomes (or lack thereof)”.

[28] (p. 873)

The tensions between governments’ simultaneous responsibility for public safety and for the support of industry may be difficult to reconcile in a neoliberal political environment. At the present time, it appears that the internationalization of neo-liberal corporate bias—that is, cross-jurisdiction regulation in support of the industry’s economic standingprevails [54] And, where Pollack provides a compelling description of the global position of the pharmaceutical industry as ‘newly fragile’, her arguments highlight the interests of the industry in retaining and bolstering its capital in the field of medicines [55]) (Figure 1m). The consequences for medicine users—taken up by some in recent research [56], will require on-going monitoring.

Given the forms of capital governments wield—particularly, in possessing social and symbolic capital leveraged to regulate the pharmaceutical industry as well as the professions of medicine and pharmacy (Figure 1g,k,n), governments establish the ‘rules of the game’ in the field of medicines, that construct particular and varied consequences for medicine users/non-users.

### 2.4. The Pharmaceutical Industry

As suggested above, the pharmaceutical industry holds high economic capital in the field of medication. Current intellectual property legislation patent protection and drug regulatory laws—in balance—protect the industry’s commercial/capital rights and limit its responsibilities to the public (a recent headline is illustrative of this position [57]). Its favourable economic position balances on its cultural, and symbolic (even moral) capital—depicted in its appeal for recognition and freedom to invest in the discovery of cures for the causes of human suffering, as reflected in the following excerpts:

“The International Federation of Pharmaceutical Manufacturers and Associations advocates policies that encourage discovery of and access to life-saving and life-enhancing medicines to improve the health of people everywhere” [58], and

“PhRMA, the Pharmaceutical Research and Manufacturers of America, represents the country’s leading biopharmaceutical researchers and biotechnology companies. Our members are committed to finding tomorrow’s cures and treatments for some of the most serious diseases such as Cancer, Alzheimer’s Disease, Cystic Fibrosis and Parkinson’s. New medicines are an integral part of the healthcare system, providing doctors and their patients with safe and effective treatment options, extending and improving quality of life”.

[59]

In addition to a favourable manufacturing/pre-market environment, regulations pertaining to the promotion and marketing of licensed medicines are also supportive of the pharmaceutical industry (Figure 1m). The industry’s access to and efforts to influence prescribing by physicians was discussed previously. However, the industry also has an interest in influencing the public’s views of its products. While patients might be conceived as relatively passive objects in health care—at least as reflected in the legal rights of doctors to enable or restrict patients’ access to prescription medicines—the pharmaceutical industry recognizes the economic capital wielded by patients/consumers, and has established sophisticated methods to influence their active involvement in their own health surveillance and in making medication choices [60,61]. The industry’s efforts to influence the public’s use of its products is reflected both in direct-to-consumer advertising (DTCA)—allowed by law in New Zealand and the United States, and in the immense non-prescription and alternative medicines markets where advertisements target users, who are conceived as active, independent agents [62,63] (Figure 1o).

The broad prohibition of DTCA, including in Canada and the European Union member countries, is intended to be a health protection measure linked to prescription-only medicines, in recognition of their potential serious harmful effects, inadequate knowledge of their potential effects, or because of the complex or serious health problems they are used to treat and the heightened vulnerability of users to inappropriate or unnecessary medication use [64] (Figure 1k). DTCA prohibitions reflect the view that industry cannot be a reliable source of patient information due to the inherent financial conflicts of interest [53]. However, as detailed in a recent empirical study, the public provision of information about medicines on the internet reveals it to be sub-optimal—to be inadequately taken up by European countries’ regulatory agencies, thus impeding the public’s access to information about new (and old) medicines [52]. In the European Union, persisting pressure from the European Commission’s Trade and Economic Development body endorsing the right of the pharmaceutical industry to provide information about its products to the public has resulted in recent concessions made to the industry. An important part of the process appeared to be the redirection of the proponents’ message from the right of industry to provide information, to the right of patients to have unimpeded access to it [53] (p. 770) (Figure 1p). It is important to note, however, that medication promotions directed at potential users does not mean that those messages are uncritically taken up: The user can and does resist [60] (Figure 1q).

The industry has wide leeway—given the absence of restrictions—to provide funding to patient/disease advocacy groups whose interests align with the industry on several levels pertaining to the promotion of medicines in health care: the promotion of the newest medicines and the need for unrestricted access to them; the need for acceleration of drug testing; the promotion of the benefit of industry advertisements to ‘inform and empower’ the public about health care management [65] (Figure 1o). Critics contend that targeted marketing sought by the industry—actively seeking partnerships that provide it explicit tangible benefits—undermines such groups’ autonomy and ability to assess the credibility of their sponsor’s claims [65,66].

The success of the industry for the industry is readily apparent—reflected in monies invested and profits earned [67,68]. However, what can be made of the industry’s success for the public—for users seeking access to safe, effective and affordable medicines?

### 2.5. Medicine Users

As the recipients of the benefits or harms of medicines, who legitimize health professionals’ recommendations when they accept them, and who support the pharmaceutical economy, it can be argued that users are the most important of agents in the field of medicines. As elaborated above, users are influenced by the interests and actions of other actors/agents, but they also exert considerable influence in the field. In this section, I discuss users of medicines, and their sources of capital in the field of medications.

That medicine-prescribing is normative in health care, and medicines are consumed in increasingly large quantities reflects the economic capital that medicine users embody—as the direct or indirect purchasers and consumers of medicines. In addition, as users or resisters of medicines, users’ cultural capital is expressed in the form of lay-knowledge about medicines that users assume in negotiating their uses.

Initially, perspectives on the medicine-user appeared in research undertaken in response to a massive and accumulating body of clinical research on patient ‘non-compliance’ or ‘non-adherence’, and served to de-centre its underlying assumptions of medical control and authority over medicines and its depiction of the ‘problematic (*i.e.*, non-adherent) patient’ [69]. Research on lay-perspectives on medicines has come to show users to be thoughtful, considered agents of medicine use and consumption, who take responsibility for medication decisions about the suitability of medicines for specific conditions and contexts [13,17,70,71,72,73,74,75,76,77,78] (Figure 1h).

Research has revealed users’ ambivalence about medicines to be a central theme, one that captures the reasoned view of medicines as both helpful and harmful, as potentially ‘remedy and poison’ [79], as producing not only physical but emotional side effects [80], and as requiring, among lay-users, the practice of testing medicines and managing their effects as they are integrated into lives over time [13]. As shown in a synthesis of thirty-seven qualitative studies of medicine taking, concern over such issues as dependence, tolerance, addiction, the potential harm from taking medicines on a long-term basis, the possibility of medicines masking other symptoms, or harms related to stigma of chronic medicine use accounts for users’ ambivalence toward and resistance to medicine-use [36]. Adding substantially to what was missing from the ‘adherence’ literature—that user compliance or adherence to medication-use directives does not eliminate possible unintended and negative effects of using medicines; that for some, the financial costs of medicines are prohibitive; that some medicines are unnecessary; that using medicines modifies one’s self concept, *etc.* this body of research established users’ cultural capital in this field.

The legitimacy given the user perspective in the field of medications—reflected in evolving practice philosophies of the professions of medicine (‘patient-focused care’) and pharmacy (‘pharmaceutical care’), and these professions’ concerns with the practitioner-patient relationship, the impacts of ‘shared decision-making’ and ‘collaborative care’ [38,81]—demonstrates the social and symbolic capital embodied in a users’ perspective on medicines (Figure 1h,r). Further, clinical and administrative research documenting over-prescribing to older adults [82,83], the demonstrated anticholinergic properties and associated iatrogenic effects of many medications prescribed to this population [84,85], as well as indications of anti-therapeutic applications of medications in institutional settings [86] illustrate the alignment of clinical- with lay-concerns about the potential harms of medicines.

A challenge to biomedical dominance or hegemony [87] over medicines’ place in health care, in a neo-liberal environment, users’ assumptions of individual responsibility for health and their critical health-seeking practices related to medicine-use represent a kind of counter-hegemony [88] to biomedical authority. Medicines have come to feature prominently in the consumer health movement where “the pursuit of health…has become one of the more salient practices of contemporary life…” [89] (p. 404), and where it is assumed that patients want to/should be informed participants in medical decision-making, and that such participation will be empowering to them [90]. Consumers can hold medical practitioners to account, both adopting and making demands of conventional biomedicine [91], and challenging its limitations [92]. It is notable, however, thatsome research challenges the view of consumerism as empowering for patients, charging that patient-consumerism reflects ‘involuntary autonomy’ [90], or that it reinforces medical dominance, medicalization and pharmaceuticalization, *i.e.*, such as when chronically ill patients adopt and emulate medical experts’ perspectives and practices [91,92,93]).

Reflecting the lay/user counter-hegemony is the recognition of lay environments—households, traditional- and virtual-communities—as the typical settings of medicines negotiations and use [14,91,94,95,96] (Figure 1s,t). Dew *et al.* [14] depict households as unique sites of ‘truth production’ about medicines where lay beliefs, experiences and practices take precedence over, or at least alongside, the authority once taken-for-granted by the professions of medicine and pharmacy. Drawing on the Latourian notion of hybridity, Dew *et al.* argue that households, as settings of medication practice, mix and reassemble the standardized practices of medicine where, “lay beliefs and practices are inherently a challenge to the power of medicine, in particular because they are not readily visible, and not readily disciplined” [14] (p. 29).

While the perspectives of Dew and colleagues suggest that the invisibility of consumer choices and uses of medicines to conventional authority figures is a source of users’ power in the field, highly visible public campaigns led by patients, patient advocacy groups and non-governmental organizations (NGOs) have been successful in gaining access to medicines for large, often politically marginalized populations. In particular, the global health consumer movement for persons living with HIV and AIDS (PLHA) has proven highly successful [97,98]—enlisting a type of symbolic/moral capital based on ‘therapeutic citizenship’ [99] or biosociality [100]—where access for a collective of vulnerable persons is achieved in a situation where access for an individual is unattainable. Summarizing the successes in this area, one legal observer noted:

“one-by-one, activists have attacked structural and legal barriers to access and have advocated for new institutional arrangements and new practices that might make treatment a reality…by promoting generic competition, they have pushed down the costs of ARVs in developing countries ….and have raised global resources for AIDS…relying on the rhetoric of a human right to health, to access to medicines, and to life itself…they have effectively removed structural impediments and leveraged resources to actually increase access to medicines on the ground”….

[101] (p. 245)

This author points to the power advocacy groups/NGOs have had in demanding that government regulators yield to populations’ needs, by modifying the regulations under which the pharmaceutical industry produces and competes for markets (Figure 1u,v).

In sum, in the field of medications, users’ access to economic, cultural, and social forms of capital reveals them to be favourably positioned relative to other agents—to also wield symbolic capital in their interactions with physicians and pharmacists, and the industry and government. It is notable that among users, economic and cultural capital is unequally available, and the social and symbolic capital of particularly vulnerable user groups, such as PLHA, is often only called into play by other agents on the basis a moral authority emerging from the vulnerability and powerlessness of the target group.

## 3. Conclusions

In this paper I have attempted to illuminate the complex context in which the medicine-user—the target of the pharmacy profession’s service to the public—accesses and uses medicines. An examination of the major players in the field of medications allows us to critically reflect upon the proliferation of medicines and the public’s uptake of them as a complex and continually enacted reality. This reality reflects an increasing reliance on pharmaceutical forms of health care technologies and interventions. In the neoliberal context of individual responsibilization for health, the public’s and/or individuals’ interest in maximizing the benefits of medicines and minimizing their iatrogenic effects results in users’ negotiations and contestations over whether and how to access and use medicines. The physician, pharmacist, government/regulatory bodies, industry and user roles in the field of medications are dynamic and evolving, based on differing resources (capital) used to enhance particular interests pertaining to medicines.
